# Predictors and Significance of Readmission after Esophagogastric Surgery: A Nationwide Analysis

**DOI:** 10.1097/AS9.0000000000000363

**Published:** 2024-01-26

**Authors:** Richard PT Evans, Sivesh K Kamarajah, Felicity Evison, Xiaoxu Zou, Ben Coupland, Ewen A Griffiths

**Affiliations:** From the * Department of Upper Gastrointestinal Surgery, Queen Elizabeth Hospital, Birmingham, UK; † Institute of Immunology and Immunotherapy, University of Birmingham, UK; ‡ Institute of Applied Health Research, University of Birmingham, UK; § Health Data Science Team, Research and Development, Queen Elizabeth Hospital, Birmingham, UK.

**Keywords:** esophagectomy, gastrectomy, readmission

## Abstract

**Objective:**

The aim of this study is to identify risk factors for readmission after elective esophagogastric cancer surgery and characterize the impact of readmission on long-term survival. The study will also identify whether the location of readmission to either the hospital that performed the primary surgery (index hospital) or another institution (nonindex hospital) has an impact on postoperative mortality.

**Background:**

Over the past decade, the center-volume relationship has driven the centralization of major cancer surgery, which has led to improvements in perioperative mortality. However, the impact of readmission, especially to nonindex centers, on long-term mortality remains unclear.

**Methods:**

This was a national population-based cohort study using Hospital Episode Statistics of adult patients undergoing esophagectomy and gastrectomy in England between January 2008 and December 2019.

**Results:**

This study included 27,592 patients, of which overall readmission rates were 25.1% (index 15.3% and nonindex 9.8%). The primary cause of readmission to an index hospital was surgical in 45.2% and 23.7% in nonindex readmissions. Patients with no readmissions had significantly longer survival than those with readmissions (median: 4.5 *vs* 3.8 years; *P* < 0.001). Patients readmitted to their index hospital had significantly improved survival as compared to nonindex readmissions (median: 3.3 *vs* 4.7 years; *P* < 0.001). Minimally invasive surgery and surgery performed in high-volume centers had improved 90-day mortality (odds ratio, 0.75; *P* < 0.001; odds ratio, 0.60; *P* < 0.001).

**Conclusion:**

Patients requiring readmission to the hospital after surgery have an increased risk of mortality, which is worsened by readmission to a nonindex institution. Patients requiring readmission to the hospital should be assessed and admitted, if required, to their index institution.

## Abstract 2

Mini abstract:

A national population-based cohort study of 27,000 patients examined the impact of readmission after elective esophagogastric surgery for cancer. Patients requiring readmission to the hospital after surgery have an increased risk of mortality, which is worsened by readmission to a nonindex institution. Patients requiring readmission to the hospital should be assessed and admitted, if required, to their index institution.

## INTRODUCTION

Esophagectomy and gastrectomy are associated with significant morbidity and mortality.^1,2^ Evidence has shown that in complex elective cancer surgery, there is an inverse relationship between hospital volume and surgical mortality, which has driven the centralization of esophagogastric surgical services in the UK.^3–5^ The development and coordination of hub and spoke services have evolved to limit the number of low-volume units, which has led to improved perioperative outcomes for patients. There have been concerns, however, that smaller peripheral hospitals that do not have a centralized resectional service subsequently lose the capability and experience to manage complex esophagogastric conditions and complications after surgery. In centralized services, pathways are built to minimize postoperative complication and readmission risk, yet despite this complications are frequent after esophageal and gastric resection with overall complication rates after esophagectomy and gastrectomy commonly as high as 60 and 30%, respectively.^6,7^

Readmission after esophagectomy occurs in 10% to 14% of cases, and patients who are not readmitted to their operative center often require transfer. Readmission after gastrectomy has been shown to be equally high at around 12%.^8^ Clinical cause for readmission and status of the patient, local expertise, and geographic factors may all influence the location of readmission and the subsequent need and ability to facilitate transfer. Fragmentation of care and readmission to a nonindex hospital has been demonstrated to be associated with increased mortality after major cancer surgery.^9^ Recognition and management of complications have been identified as key areas for improvement to minimize postoperative mortality. Concentrated expertise in centralized services has been associated with minimizing “failure to rescue.”^10^ There is, however, limited information on the predictors and significance of readmission after elective esophagogastric surgery. Furthermore, the proportion and impact of readmission to a center that does not perform esophagectomy or gastrectomy is unknown.

The aim of this study is to identify risk factors for readmission and determine whether readmission impacts postoperative mortality. The secondary aim of this study is to determine whether the location of readmission to either the hospital that performed the primary surgery (index hospital) or another institution has an impact on postoperative mortality.

## METHODS

### Inclusion, Exclusion Criteria, and Data Source

This was a national population-based cohort study of adult patients undergoing National Health Service (NHS)-funded elective esophagectomy and gastrectomy for cancer in England between 2008 and 2019, using the Hospital Episodes Statistics Database (HES) for upper-gastrointestinal cancer. Patient outcome data including mortality was included until 12/2020, to ensure all patients had at least 12 months of follow-up. This study was registered with the local Clinical Audit Department (Clinical Audit Registration and Management System number 15126). Data were used in line with the data sharing agreement with NHS Digital.

HES data includes clinical information about diagnoses and operations patient information, such as age group, gender, and ethnicity, administrative information, such as dates and methods of admission and discharge, and geographical information, such as where patients are treated and the area where they live. Patient-specific HES identifiers enable patients to be tracked over multiple hospital admissions. Diagnoses are recorded using the International Classification of Disease version 10 codes, from which clinical comorbidity can be identified. NHS healthcare providers collect administrative and clinically relevant information to facilitate institute financial remuneration. Digital recording is performed by administrative staff within an institute coding department from various clinical sources, including discharge summaries, and radiological and operative databases. Mortality data is acquired via HES-linked Office for National Statistics data death data.

Patients with missing age or sex were excluded from analysis as these fields were used historically to derive the HES identifier. Patients who were not resident in England were also excluded from the analysis, as their follow-up may occur elsewhere. Elective readmission was excluded from the analysis. If, for any reason, a patient underwent multiple gastrectomies or esophagectomies only the first procedure was kept. Volume was defined as the average annual number of procedures per hospital and was calculated before exclusions were made. A modified version of the Charlson Comorbidity Index was used to measure comorbidity, excluding cancer and metastatic cancer.^11^ The Indices of Multiple Deprivation were used to describe socio-economic deprivation, for admissions between 2008 and 2010 the 2007 version of Index of Multiple Deprivation was used, for admissions between 2011 and 2014 the 2010 version was used and for admissions after 2014, the 2015 version was used.

### Operations

Operative procedures were identified by the Office of Population Censuses and Surveys Classification of Surgical Operations and Procedures 4th revision codes. Esophagectomy codes included G01, G02, and G03. Gastrectomy codes included G27 and G28. Minimal access surgery was defined by codes Y74 and Y75.

### Explanatory Variable

The main exposure variable was readmission within 90 days. This was inclusive of unplanned admissions only. Index hospital was defined as the hospital in which the primary surgery was performed and nonindex hospital was defined as any other hospital. If a patient was initially readmitted to a nonindex trust but on the day of readmission or on the following day they were transferred to the index trust, this was recorded as readmission to the index trust.

### Statistical Analysis

Categorical variables are shown as counts and percentages, continuous as medians and inter-quartile ranges. Univariable analysis of categorical variables used chi-square tests and Kruskall–Wallis for continuous variables. Multivariable logistic regression was used to analyze readmissions and deaths within 90 days, the variables to be included were decided on a priori based on clinical knowledge. Overall patient survival was analyzed using Kaplan–Meier plots starting at 90 days postdischarge and excluding anyone who died in this period. Multivariable Cox analysis was performed with readmission being treated as a time-dependent variable. Proportional hazards were assessed via Schoenfeld residuals, as a result of this assessment, the Cox analysis was restricted to 5 years postdischarge. Analysis was performed on the overall cohort and also on the sub-cohorts of procedure type. Statistical analysis was performed in Stata Statistical Software: Release 15 SE (StataCorp LLC Texas). The approximate duration of car journeys from the center of the lower super output area that a patient lives into the site of their admission or readmission was calculated using the R package OSRM.^12^ Readmission and survival plots were analyzed in R using package success (https://CRAN.R-project.org/package=success).^13^

## RESULTS

### Clinicopathologic Characteristics

From January 2008 to December 2019, 27,591 patients underwent elective esophagectomy or gastrectomy in England. This included 18,337 who underwent esophagectomy and 9254 who underwent either total or partial gastrectomy (total n = 4659, partial = 4595, Supplemental Tables 1 and 2, http://links.lww.com/AOSO/A271). Readmission after esophagectomy occurred in 27.6% of cases, of which 16.3% were readmitted to their index hospital (Table 1). Totally, 31.7% of esophagectomies were performed using minimally invasive techniques, and reoperation during index admission occurred in 4.4% of cases. Ninety-day mortality was 4.4%, and 5-year mortality was 51.6%. In total 13.0% of gastrectomies were performed using minimally invasive techniques, and reoperation during index admission occurred in 3.4% of cases. Ninety-day mortality was 4.1%, and 5-year mortality was 45.6%.

**TABLE 1.** Clinicopathologic Characteristics Associated With Readmissions in Patients Undergoing Esophagectomy

		No Readmission	Index Readmission	Nonindex Readmission	*P*
	*n*	13454 (73.4%)	2994 (16.3%)	1889 (10.3%)	
Patient age
	Median age (IQR)	66 (59–72)	65 (58–72)	66 (59–72)	0.003*
	18–56	2317 (17.2%)	597 (19.9%)	337 (17.8%)	0.014
	57–64	3587 (26.7%)	811 (27.1%)	472 (25.0%)	
	65–69	2892 (21.5%)	600 (20.0%)	399 (21.1%)	
	70–75	3007 (22.4%)	636 (21.2%)	451 (23.9%)	
	76+	1651 (12.3%)	350 (11.7%)	230 (12.2%)	
Sex	Male	10649 (79.2%)	2284 (76.3%)	1455 (77.0%)	0.001*
	Female	2805 (20.9%)	710 (23.7%)	434 (23.0%)	
Ethnicity	White	12706 (94.4%)	2806 (93.7%)	1833 (97.0%)	<0.001*
	Asian	108 (0.8%)	50 (1.7%)	15 (0.8%)	
	Nonwhite Non-Asian	67 (0.5%)	19 (0.6%)	6 (0.3%)	
	Other	573 (4.3%)	119 (4.0%)	35 (1.9%)	
Deprivation quintiles	1	2056 (15.3%)	513 (17.1%)	315 (16.7%)	0.002*
	2	2523 (18.8%)	540 (18.0%)	383 (20.3%)	
	3	2923 (21.7%)	609 (20.3%)	426 (22.6%)	
	4	3051 (22.7%)	665 (22.2%)	426 (22.6%)	
	5	2901 (21.6%)	667 (22.3%)	339 (18.0%)	
Charlson score (without cancer)	0	8750 (65.0%)	1830 (61.1%)	1135 (60.1%)	<0.001*
	1–4	2706 (20.1%)	679 (22.7%)	442 (23.4%)	
	5+	1998 (14.9%)	485 (16.2%)	315 (16.5%)	
Volume grouping (use the average annual operation volume of hospital to split the cohorts equally)	<=42	1809 (13.5%)	404 (13.5%)	186 (9.9%)	<0.001*
	43–64	2781 (20.7%)	612 (20.4%)	452 (23.9%)	
	65–74	3065 (22.8%)	716 (23.9%)	351 (18.6%)	
	75–97	3034 (22.6%)	625 (20.9%)	500 (26.5%)	
	>97	2765 (20.6%)	637 (21.3%)	400 (21.2%)	
Reoperation (within the same admission)	Yes	670 (5.0%)	147 (4.9%)	72 (3.8%)	0.085
Minimal access surgery	Yes	4211 (31.3%)	1002 (33.5%)	593 (31.4%)	0.068
Length of stay	Median length of stay (IQR)	14 (11–21)	15 (11–21)	15 (11–22)	0.129
90-day mortality	Yes	619 (4.6%)	113 (3.8%)	81 (4.3%)	0.132
5-year mortality	Yes	6910 (51.4%)	1535 (51.3%)	1015 (53.7%)	0.144

* *P* values of ≤0.05.

IQR indicates interquartile range.

The primary cause of readmission to an index hospital was surgical in 45.2% of cases whereas the primary cause for readmission to a nonindex hospital was surgical in 23.7% of cases (Tables 2 and 3). The cause of readmission did not significantly impact overall survival for esophagectomy and gastrectomy (Supplemental Figure 1 and 20, http://links.lww.com/AOSO/A272.

**TABLE 2.** Clinicopathologic Characteristics Associated with Readmissions in Patients Undergoing Gastrectomy

		No Readmission	Index Readmission	Non-index Readmission	*P*
	*n*	7216 (78.0%)	1227 (13.3%)	812 (8.9%)	
Patient age	Median age (IQR)	71 (62–77)	70 (60–76)	71 (62–77)	<0.001*
	18–56	1094 (15.2%)	245 (20.0%)	126 (15.5%)	0.001*
	57–64	1046 (14.5%)	185 (15.1%)	115 (14.2%)	
	65–69	1069 (14.8%)	181 (14.8%)	104 (12.8%)	
	70–75	1697 (23.5%)	275 (22.4%)	212 (26.1%)	
	76+	2310 (32.0%)	341 (27.8%)	255 (31.4%)	
Sex	Male	4813 (66.7%)	780 (63.6%)	539 (66.4%)	0.1
	Female	2403 (33.3%)	447 (36.4%)	273 (33.6%)	
Ethnicity	White	6326 (87.7%)	1064 (86.7%)	734 (90.4%)	0.006
	Asian	253 (3.5%)	64 (5.2%)	22 (2.7%)	
	Non-White non-Asian	292 (4.1%)	49 (4.0%)	33 (4.1%)	
	Other	345 (4.8%)	50 (4.1%)	23 (2.8%)	
Deprivation quintiles	1	1545 (21.4%)	265 (21.6%)	179 (22.0%)	0.571
	2	1425 (19.8%)	217 (17.7%)	165 (20.3%)	
	3	1483 (20.6%)	267 (21.8%)	170 (20.9%)	
	4	1430 (19.8%)	238 (19.4%)	166 (20.4%)	
	5	1333 (18.5%)	240 (19.6%)	132 (16.3%)	
Charlson score (without cancer)	0	4343 (60.2%)	700 (57.1%)	471 (58.0%)	0.193
	1–4	1449 (20.1%)	267 (21.8%)	181 (22.3%)	
	5+	1424 (19.7%)	260 (21.2%)	160 (19.7%)	
Volume grouping (use the average annual operation volume of hospital to split the cohorts equally)	<=42	1164 (16.1%)	221 (18.0%)	91 (11.2%)	<0.001*
	43–64	1563 (21.7%)	234 (19.1%)	228 (28.1%)	
	65–74	1444 (20.0%)	283 (23.1%)	137 (16.9%)	
	75–97	1480 (20.5%)	227 (18.5%)	192 (23.7%)	
	>97	1565 (21.7%)	262 (21.4%)	164 (20.2%)	
Reoperation (within the same admission)	Yes	257 (3.6%)	46 (3.8%)	12 (1.5%)	0.006
Minimal access surgery	Yes	948 (13.1%)	165 (13.5%)	89 (11.0%)	0.19
Length of stay	Median length of stay (IQR)	12 (9–16)	13 (10–18)	12 (10–17)	<0.001*
90-day mortality	Yes	286 (4.0%)	52 (4.2%)	41 (5.1%)	0.322
5-year mortality	Yes	3180 (44.1%)	628 (51.2%)	410 (50.5%)	<0.001*

* *P* values of ≤0.05.

IQR indicates interquartile range.

**TABLE 3.** Primary Cause of Readmission to Hospital

Esophagectomy		Index Readmission		Nonindex Readmission	
		No of Patients	%	No of Patients	%
Primary diagnosis	Surgical				
	** **Diagnosis 1	Malignant neoplasm: esophagus	171	Malignant neoplasm: esophagus	102
	** **Diagnosis 2	Postprocedural disorders of digestive system*	138	Other	45
	** **Diagnosis 3	Nausea and vomiting*	125	Nausea and vomiting*	39
	** **Diagnosis 4	Other	124	Mechanical complication of gastrointestinal prosthetic devices, implants, and grafts	36
	** **Diagnosis 5	Dysphagia	120	Abdominal pain/malignant neoplasm	30
	Nonsurgical				
	** **Diagnosis 1	Other	347	LRTI	255
	** **Diagnosis 2	LRTI	247	Other	240
	** **Diagnosis 3	Nausea and vomiting*	102	Nausea and vomiting*	111
	** **Diagnosis 4	Postprocedural disorders of digestive system*	97	Chest Pain	67
	** **Diagnosis 5	Pleural effusion	83	Pleural effusion	66
Gastrectomy		Index Readmission		Nonindex Readmission	
		No of Patients	%	No of Patients	%
Primary diagnosis	Surgical				
	** **Diagnosis 1	Malignant neoplasm: stomach	88	Abdominal pain	35
	** **Diagnosis 2	Postprocedural disorders of GI system/infection following a procedure	73	Infection following a procedure	30
	** **Diagnosis 3	Other	63	Malignant neoplasm stomach	27
	** **Diagnosis 4	Abdominal pain	57	Nausea and vomitting*	19
	** **Diagnosis 5	Nausea and vomiting*	49	Other	16
	Nonsurgical				
	** **Diagnosis 1	Other	155	Other	153
	** **Diagnosis 2	GI Infection	35	LRTI	52
	** **Diagnosis 3	Postprocedural disorders of GI system	34	Renal complications	35
	** **Diagnosis 4	Renal complications	32	GI infection/infection/sepsis source not specified	32
	** **Diagnosis 5	LRTI/malignant neoplasm: stomach	31	Nausea and vomitting*	25

* Nausea and vomiting admitted under surgery were identified as a “surgical readmission”, whereas if it was admitted under internal medicine it was identified as “nonsurgical”.

GI indicates gastrointestinal; LRTI, lower respiratory tract infection.

Median duration of car journey to the index procedure hospital was 25.8 minutes (15.7–41.5). Time taken to attend hospital for readmission was significantly shorter when presenting to a nonindex hospital as compared to an index hospital (*P* < 0.001, nonindex site = 13 minutes [8.5–21.5], index site =19 minutes [11.8–29.8]). Patients who were readmitted to a nonindex institution were significantly more likely to go to a nonteaching hospital (*P* < 0.001, nonindex site readmission, teaching hospital = 1,122 [41.5%] non-teaching = 1,579 [58.5%]).

## FACTORS ASSOCIATED WITH READMISSION

### Overall Readmission and Adjusted Analysis Data

**FIGURE 1.**
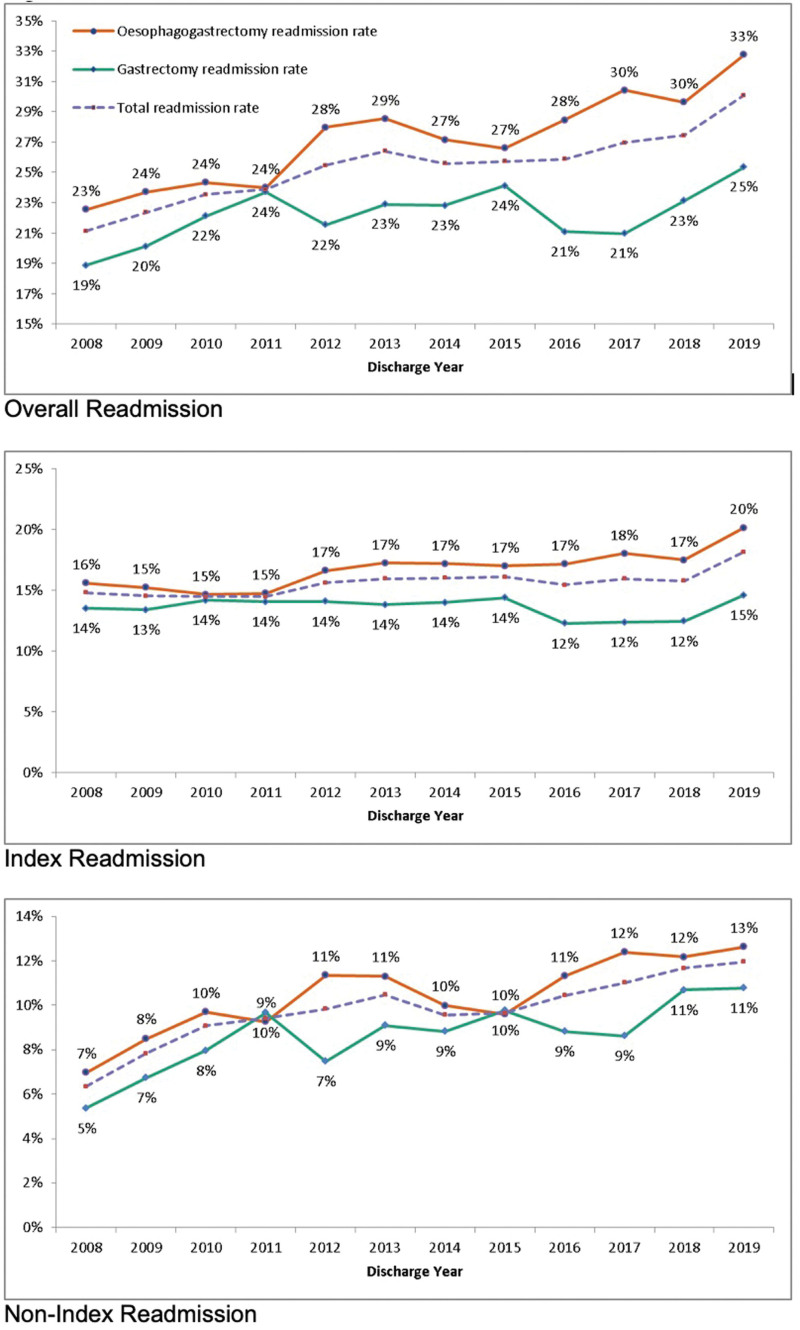
Readmission rates over time. Overall Readmission. Index Readmission. NonIndex Readmission

Overall readmission rates have increased steadily across the study period from 21% in 2008 to 30% in 2019 (Fig. 1), with index readmission from 15% to 18% and nonindex readmission from 6% to 12% over the same time period. Overall, index and nonindex readmission rates increased in both esophagectomy and gastrectomy (Fig. 1).

### Esophagectomy Only

Patients undergoing esophagectomy were more likely to be readmitted if they were female (odds ratio [OR], 1.17; *P* < 0.001) or of Asian ethnicity (OR, 1.58; *P* = 0.005) (Supplemental Table 1, http://links.lww.com/AOSO/A271). Patients with increasing comorbidity were at greater risk of readmission (Charlson 1–4: OR, 1.24; *P* < 0.001 and Charlson 5+: OR, 1.30; *P* < 0.001). Reoperation and use of minimally invasive techniques did not impact readmission rates (OR, 1.02; *P* = 0.780 and OR, 1.07; *P* = 0.057). Hospital volume has a mixed impact on readmission rates with centers performing 43–64 and >97 esophagectomies demonstrating higher readmission rates (43–64: OR, 1.17; *P* = 0.011 and >97 OR, 1.15; *P* = 0.025, Supplemental Figure 3, http://links.lww.com/AOSO/A274). Readmission rate was unaffected for centers performing 65–74 and 75–96 esophagectomies.

### Gastrectomy Only

Patient sex did not impact readmission rates for patients undergoing gastrectomy (OR, 1.09; *P* = 0.110) (Supplemental Table 4, http://links.lww.com/AOSO/A271). Ethnicity and deprivation scores equally did not impact readmission rates. Patients with increased comorbidity were at greater risk of readmission (Charlson 1–4: OR, 1.59; *P* = <0.021 and Charlson 5+: OR, 1.23; *P* = <0.002). Reoperation and use of minimally invasive techniques did not impact readmission rates (OR, 1.07; *P* = 0.644 and OR, 0.93; *P* = 0.350). Readmission rate was unaffected by hospital volume (Supplemental Figure 4, http://links.lww.com/AOSO/A275).

## OVERALL SURVIVAL

### Overall Survival and Adjusted Analysis Data

**FIGURE 2.**
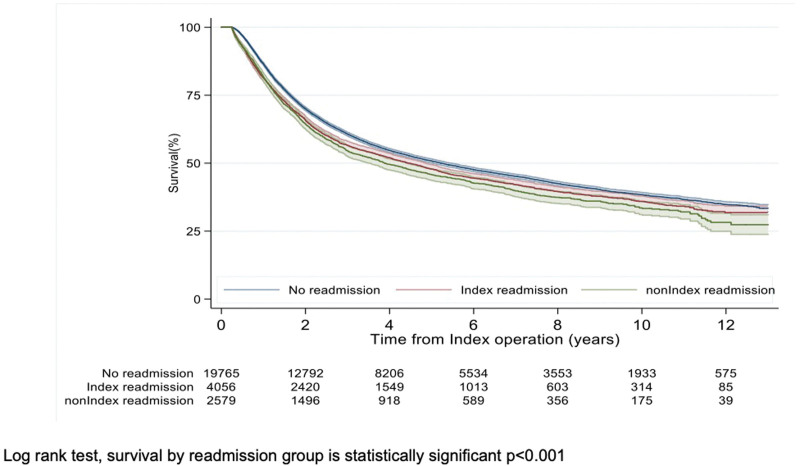
Overall survival for patients requiring readmission. Log-rank test, survival by readmission group is statistically significant *P* < 0.001.

Patients with no readmissions had significantly longer survival than those with readmissions (median [years]: 4.5 *vs* 3.8; *P* < 0.001). Of patients who were readmitted, those readmitted to their index hospital had significantly improved survival (Fig. 2). In adjusted analyses, patients requiring index and nonindex readmission had significantly shorter survival than those without readmission (median (years): 3.3 *vs* 4.7; *P* < 0.001).

### Esophagectomy Only

Overall survival after esophagectomy was improved in women (hazard raio [HR], 0.73; *P* < 0.001) (Table 4). Survival was significantly reduced for those with increasing age. Patients in the upper quintile of age, 76+ years old demonstrated the worst 90-day mortality (HR, 1.451; *P* < 0.001). Patients with increasing comorbidity were not at greater risk of reduced survival (Charlson 1–4: HR, 1.10; *P* = 0.174 and Charlson 5+: HR, 1.10; *P* < 0.269). Reoperation was not associated with significantly reduced overall survival (HR, 1.12; *P* < 0.379) whereas use of minimally invasive techniques was associated with improved survival (HR, 0.86; *P* < 0.021). Long-term survival was affected by center volume (center vol. 75–97; HR, 0.91; *P* = 0.369; center vol. >97; HR, 1.06; *P* = 0.597; Supplemental Figure 5, http://links.lww.com/AOSO/A276). Patients readmitted with recurrence were identified to have worse overall survival (readmission cause, malignant neoplasm of the esophagus HR, 1.29; *P* = 0.012). Cause of readmission had little impact on overall survival (Supplemental Figure 1, http://links.lww.com/AOSO/A272).

**TABLE 4.** Multivariate Analysis of Factors Associated with Overall Survival Postsophagectomy

	Hazard Ratio	*P* >z	(95% CI)	
Gender				
Male	1			
Female	0.73	<0.001*	0.64	0.84
Age groups (years)				
18–56	1			
57–64	1.11	0.229	0.94	1.31
65–69	1.16	0.117	0.96	1.39
70–75	1.18	0.073	0.98	1.42
76+	1.45	0.001*	1.17	1.79
Ethnicity				
White	1			
Asian	1.10	0.69	0.69	1.74
Non-White non-Asian	0.63	0.312	0.26	1.54
Other	0.98	0.889	0.70	1.35
Deprivation quintiles				
1	1			
2	0.81	0.022*	0.67	0.97
3	0.84	0.051*	0.70	1.00
4	0.87	0.121	0.73	1.04
5	0.79	0.013*	0.66	0.95
Charlson Comorbidity Index				
0	1			
1–4	1.10	0.174	0.96	1.26
5+	1.10	0.269	0.93	1.29
Reoperation–No	1			
Reoperation–Yes	1.12	0.379	0.87	1.45
Minimal access–No	1			
Minimal access–Yes	0.86	0.021*	0.76	0.98
Hospital volume groups				
<=42	1			
43–64	0.98	0.859	0.80	1.20
65–74	0.99	0.927	0.81	1.21
75–97	0.91	0.369	0.75	1.11
>97	1.06	0.597	0.86	1.29
Readmission organization				
Readmission to index center	1			
Readmission to nonindex center	1.08	0.19	0.96	1.22
Readmission reason				
LRTI	1			
Abdominal pain	1.12	0.365	0.88	1.43
Dysphagia	0.84	0.207	0.65	1.10
Infection following a procedure NEC	1.04	0.787	0.80	1.34
Malignant neoplasm of esophagus	1.29	0.012*	1.06	1.57
Mechanical complication of GI prosthetic devices	1.02	0.862	0.79	1.33
Nausea and vomiting	0.91	0.325	0.74	1.10
Other complications postmedical intervention	0.91	0.493	0.69	1.20
Pleural effusion, NEC	1.08	0.532	0.86	1.35
Postprocedural disorders of digestive system	1.04	0.685	0.85	1.28

* *P* values of ≤0.05.

GI indicates gastrointestinal; LRTI, lower respiratory tract infection; NEC, not elsewhere classifiable.

### Gastrectomy Only

Overall survival after gastrectomy was affected by patient sex (female HR, 0.94; *P* < 0.558) (Table 5). Survival was not impacted by patient’s age at the time of surgery. Deprivation did not impact survival in patients undergoing gastrectomy. Severe comorbidity identified as a Charlson >5 did not correlate with worse overall survival, patients with a Charlson score (CS) of 1–4 were equally unaffected (CS 5+ HR, 1.06; *P* < 0.645; CS 1–4; HR, 1.01; *P* = 0.909). Reoperation did not affect overall survival (HR, 1.39; *P* < 0.184) neither did the use of minimally invasive techniques (HR, 1.04; *P* < 794). Center volume did significantly alter overall survival for patients undergoing gastrectomy (Supplemental Figure 6, http://links.lww.com/AOSO/A277). The cause of readmission had little impact on overall survival (Supplemental Figure 2, http://links.lww.com/AOSO/A273).

**TABLE 5.** Multivariate Analysis of Factors Associated with Overall Survival Post Gastrectomy

	Hazard Ratio	*P* >z	(95% CI)	
Gender				
Male	1			
Female	0.94	0.558	0.78	1.15
Age groups (years)				
18–56	1			
57–64	1.05	0.783	0.76	1.44
65–69	1.25	0.167	0.91	1.70
70–75	1.13	0.419	0.85	1.50
76+	1.28	0.081	0.97	1.70
Ethnicity				
White	1			
Asian	0.47	0.009*	0.27	0.83
Non-White non-Asian	0.57	0.046*	0.33	0.99
Other	0.55	0.043*	0.30	0.98
Deprivation quintiles				
1	1			
2	0.90	0.475	0.67	1.20
3	1.11	0.44	0.85	1.46
4	0.95	0.694	0.72	1.25
5	1.20	0.206	0.90	1.61
Charlson Comorbidity Index				
0	1			
1–4	1.01	0.909	0.81	1.27
5+	1.06	0.645	0.83	1.34
Reoperation–No	1			
Reoperation–Yes	1.39	0.184	0.85	2.27
Minimal access–No	1			
Minimal access–Yes	1.04	0.794	0.79	1.37
Hospital volume groups				
<=42	1			
43–64	0.93	0.658	0.69	1.26
65–74	0.91	0.51	0.67	1.22
75–97	1.00	0.986	0.75	1.35
>97	0.91	0.528	0.67	1.22
Readmission organization				
Readmission to Index center	1			
Readmission to nonindex center	0.94	0.503	0.77	1.14
Readmission reason				
Malignant neoplasm of stomach	1			
Abdominal pain	0.79	0.141	0.57	1.08
GI infection	0.67	0.058	0.44	1.01
Infection following a procedure (NEC)	0.62	0.005*	0.45	0.87
LRTI	0.95	0.765	0.66	1.36
Nausea and vomiting	0.82	0.25	0.59	1.15
Other complications following	0.76	0.171	0.51	1.13
Postprocedural disorders	0.67	0.018*	0.49	0.93
Renal complications	0.68	0.073	0.45	1.04

* *P* values of ≤0.05.

GI indicates gastrointestinal; LRTI, lower respiratory tract infection; NEC, not elsewhere classifiable.

### 90-day Mortality

Ninety-day mortality was significantly greater in patients undergoing esophagectomy as compared to gastrectomy (OR, 0.74; *P* < 0.001) (Table 6). Patient gender and ethnicity were not predictors of 90-day mortality. Absence of deprivation (indices of multiple deprivation score 5) was protective of mortality (OR, 0.82; *P* = 0.048). Severe comorbidity identified as a Charlson >5 increased the risk of 90-day mortality, patients with a CS of 1–4 were unaffected (OR, 2.46; *P* < 0.001; OR, 1.04; *P* = 0.60). Minimally invasive surgery reduced the risk of perioperative mortality whereas reoperation was identified predict the greatest risk of 90-day mortality (OR, 0.75; *P* < 0.001; OR, 4.85; *P* < 0.001). Increasing center volume correlated with reduced risk of 90-day mortality with centers in the fifth quintile reporting the lowest risk of 90-day mortality (OR, 0.60; *P* < 0.001).

**TABLE 6.** Multivariate Analysis of Factors Associated With 90-day Mortality

90-d Mortality	Odds Ratio	*P* >z	(95% CI)	
Index op				
Esophageal surgery	1			
Gastric surgery	0.74	<0.001*	0.65	0.85
Gender				
Male	1			
Female	0.88	0.081	0.76	1.02
Age groups (years)				
18–56	1			
57–64	1.38	0.007	1.09	1.7
65–69	1.43	0.003	1.27	1.82
70–75	2.06	<0.001*	1.64	2.57
76+	2.65	<0.001*	2.11	3.33
Ethnicity				
White	1			
Asian	1.07	0.774	0.67	1.72
Non-White non-Asian	0.66	0.172	0.37	1.20
Other	1.17	0.299	0.87	1.57
Deprivation quintiles				
1	1			
2	0.93	0.440	0.76	1.12
3	0.97	0.715	0.80	1.16
4	0.91	0.329	0.75	1.10
5	0.82	0.048	0.67	1.00
Charlson Comorbidity Index				
0	1			
1–4	1.04	0.600	0.89	1.23
5+	2.46	<0.001*	2.14	2.81
Reoperation–No	1			
Reoperation–Yes	4.85	<0.001*	4.09	5.74
Minimal access–No	1			
Minimal access–Yes	0.75	<0.001*	0.65	0.87
Hospital volume groups				
<=42	1			
43–64	0.67	<0.001*	0.55	0.80
65–74	0.73	<0.001*	0.60	0.88
75–97	0.62	<0.001*	0.51	0.75
>97	0.60	<0.001*	0.49	0.73

* *P* values of ≤0.05.

## DISCUSSION

This national population-based cohort study, including >27,000 patients, demonstrated that readmission after elective esophagectomy and gastrectomy for cancer has steadily increased over the past decade with rates now as high as 30%. Nearly one-third of patients readmitted return to nonindex institutions, and one-third of readmissions arise as a result of surgical-specific complications. Critically, readmission is associated with reduced overall survival, and particularly patients readmitted to a nonindex institution have the lowest overall survival. High-volume centers and centers performing minimally invasive surgery demonstrate improved long-term survival. This data can support health service delivery and infrastructure planning to ensure patients undergoing elective esophagectomy and gastrectomy receive treatment in high-volume centers with experience in minimally invasive techniques, and should patients require readmission maximal efforts should be undertaken to ensure this is to their index center.

Progressive increases in readmission rates have been identified within this study. Increasing expertise in medicine has led to an overall improved delivery of healthcare and consequently, there is an ever-increasing aging population with increased frailty and multimorbidity. Previously perceived barriers to complex surgery such as advanced age are in isolation no longer contraindications to surgery and perhaps increasing readmission rates are a modern reflection of the population as a whole. Reduced comorbidity and absence of deprivation were identified to be protective of readmission in this study. Current evidence highlights that salvage surgery and postoperative complications were also associated with readmission.^14–16^ Reduced preoperative nutrition and evidence of sarcopenia have been shown to predict readmission in both esophagectomy and gastrectomy.^17–19^ Increasingly prehabilitation programs have been introduced to negate the impact of preoperative nutritional deficits and challenging neoadjuvant treatments. Despite this, current prehabilitation programs have not been able to reduce complication and readmission rates.^20^ Determining suitability for discharge and, in turn, preventing readmission can be clinically challenging. Increasingly machine learning techniques have been used to improve readmission risk prediction with a moderate degree of success.^21^ It is unknown, however, whether such models will have sufficient accuracy and relevance to be adopted into clinical practice.

Patients requiring readmission who are admitted to a nonindex institution have a significantly reduced overall survival.^22^ Fragmentation of care and readmission to a nonindex institution is common accounting for 20% to 33% of readmissions.^9,22,23^ These findings are supported by population-based analyses of 9,440,503 Medicare beneficiaries undergoing major surgery which examined the impact of readmission on 90-day mortality. Patients undergoing gastrectomy were not included in this study, yet 16,702 undergoing esophagectomy were included. The readmission rate for esophagectomy was 21.9%, of which 66.8% were readmitted to their index institution. Overall and for oesophagectomy patients specifically, mortality was reduced for patients admitted to their index institution. Analysis of patients readmitted after gastrectomy shows that patients readmitted with a surgical complication have an increased mortality rate when readmitted to a nonindex institution.^24^ Improvements in short-term outcomes may be attributable to greater clinician experience enabling earlier recognition of complications requiring intervention. Institutional variances may also exist in the aggressiveness of care and end-of-life decisions.^23^ Centralization has enabled improved 24-hour access to interventional radiology, theaters, and dedicated oesophagogastric surgeons on-call rotas. Variation in long-term survival as a result of fragmentation in care may again be attributable to access to clinicians with a greater experience in diagnostics of post-op complications. More minor complications, such as recurrent chest infections or nutritional complications that are not managed adequately may have long-term consequences.^23^ Failure of active nutritional assessment and intervention has been shown to reduce survival in patients with pancreatic adenocarcinoma.^25^ Index hospitals were shown to provide improved nutritional assessment and pancreatic enzyme replacement which was associated with improved long-term survival.^26^

Textbook outcome after both esophagectomy and gastrectomy has been shown to be associated with improved long-term survival.^27^ Centralization has improved patient outcomes in esophagectomy and gastrectomy, however, despite this, there has been a progressive increase in readmission rates during the study period. Results from the Dutch Upper-Gastrointestinal Cancer Audit have shown progressive improvement over time in surgical standards with increasing lymph node yields, increased rates of minimally invasive surgery, and reduced mortality rates.^28^ Complication rates over the same duration have, however, increased, emphasizing the importance of improved expertise in complication management that likely benefits from centralized expertise in esophagogastric surgery. In addition to complex surgery within the UK, there has also been a drive in the UK to centralize the care of complex medical problems including stroke and myocardial infarction. Hospital capacity has not increased at a rate to meet demand. This has been further complicated by COVID which has brought further pressure on elective capacity and staffing.^29^ Increasing readmission may be reflective of increased pressure for bed capacity. In addition, further funding and analysis are required to better determine how to deliver optimum care in a well-resourced centralized service particularly as it is evident that outcomes are worse for patients with surgical and nonsurgical complications readmitted to a nonindex institution.^30^ Patients requiring advice or clinical review after discharge often lack coordinated access to early assessment due to limited hospital resources and patient location. To ensure high-quality outcomes and prevent readmission improved protocols may be required which are inclusive remote monitoring.^31,32^ Wearable monitoring devices which are inclusive of heart rate, temperature, and respiratory rate are coming to the market and may provide a mechanism to create an early warning system for patients postdischarge at risk of unplanned readmission.^33^ Clinician or nurse-led remote monitoring of postsurgery patients on virtual wards may provide a mechanism for early recognition of complications. The COVID pandemic has led to improved access and willingness to use video conferencing. Studies have shown that even within the context of cancer surgery, video conferring is an acceptable method of follow-up for patients which in turn may enable improved options for centralized services to access remote patients.^34^ Combined remote monitoring and easy access to telephone and video consultation may enable early recognition of complications and repatriation to the patient’s index institution which has been identified in the study to provide improved short- and long-term outcomes for patients.

Our study has some strengths, including the large number of esophagectomy and gastrectomy patients analyzed using a population-based database. As a multi-institutional population-based study capturing patients from over 10 years, it provides a comprehensive assessment of both risk factors and implications of readmission after major surgery. The study is limited as it is retrospective and lacks some granularity that is not possible due to the nature of the data captured by HES. This is particularly important within the context of neoadjuvant and adjuvant treatment which is not available within HES. Only a very small number of patients operated in the private sector would not be included in this study. The readmission destination may also be a source of selection bias as the severity of the illness is unknown.

## CONCLUSION

Readmission rates for elective esophagectomy and gastrectomy for cancer have increased significantly over the past decade. Patients requiring readmission to the hospital after surgery have an increased risk of mortality which is further exacerbated by admission to a nonindex institution. Patients requiring readmission to the hospital should be assessed and admitted if required to their index institution.

## Supplementary Material














