# Supplementary material for: Predictors and Significance of Readmission after Esophagogastric Surgery: A Nationwide Analysis
Source: Ann Surg Open. 2024 Jan 26;5(1):e363. doi: 10.1097/AS9.0000000000000363 (PMC11175914; doi:10.1097/AS9.0000000000000363)
Supplement: Supplementary file 1 [file as9-5-e363-s001.pdf]

Supplementary Table 1 - Clinicopathologic characteristics associated with readmissions in patients undergoing total gastrectomy

		No readmission	Index readmission	Non-index readmission	*P value*
	n	3451 (74.1%)	744 (16.0%)	464 (10.0%)	
**Patient Age**	Median Age (IQR)	70 (61-76)	69 (60-75)	70 (62-76)	0.059
	18-56	575 (16.7%)	148 (19.9%)	72 (15.5%)	0.553
	57-64	524 (15.2%)	114 (15.3%)	70 (15.1%)	
	65-69	568 (16.5%)	119 (16.0%)	73 (15.7%)	
	70-75	887 (25.7%)	180 (24.2%)	117 (25.2%)	
	76+	897 (26.0%)	186 (24.6%)	132 (28.5%)	
**Sex**	Male	2529 (73.3%)	488 (65.6%)	327 (70.5%)	<0.001*
	Female	922 (26.7%)	256 (34.4%)	137 (29.5%)	
**Ethnicity**	White	3077 (89.2%)	650 (87.4%)	434 (93.5%)	0.009
	Asian	115 (3.3%)	37 (5.0%)	7 (1.5%)	
	Non-White Non-Asian	96 (2.8%)	24 (3.2%)	12 (2.6%)	
	Other	163 (4.7%)	33 (4.4%)	11 (2.4%)	
**Deprivation Quintiles**	1	722 (20.9%)	167 (22.5%)	102 (22.0%)	0.607
	2	639 (18.5%)	120 (16.1%)	90 (19.4%)	
	3	701 (20.3%)	160 (21.5%)	93 (20.0%)	
	4	741 (21.5%)	152 (20.4%)	105 (22.6%)	
	5	648 (18.8%)	145 (19.5%)	74 (16.0%)	
**Charlson score (without cancer)**	0	2133 (61.8%)	429 (57.7%)	271 (58.4%)	0.105
	1-4	691 (20.0%)	178 (23.9%)	106 (22.8%)	
	5+	627 (18.2%)	137 (18.4%)	87 (18.8%)	
**Volume grouping (use the Average annual operation volume of hospital to split the cohorts equally)**	<=42	500 (14.5%)	126 (16.9%)	50 (10.8%)	0.003
	43-64	760 (22.0%)	154 (20.7%)	121 (26.1%)	
	65-74	674 (19.5%)	164 (22.0%)	76 (16.4%)	
	75-97	762 (22.1%)	135 (18.2%)	117 (25.2%)	
	>97	755 (21.9%)	165 (22.2%)	100 (21.6%)	
**Reoperation (within the same admission)**	Yes	168 (4.9%)	*	*	0.004*
**Minimal Access Surgery**	Yes	266 (7.7%)	68 (9.1%)	39 (8.4%)	0.403
**Length of stay**	Median LOS(IQR)	13 (10-18)	13 (10-19)	13 (10-17)	0.195
**90-day mortality**	Yes	197 (5.7%)	28 (3.8%)	24 (5.2%)	0.1
**5-year mortality**	Yes	1721 (49.9%)	420 (56.5%)	251 (54.1%)	0.002

Supplementary Table 2 - Clinicopathologic characteristics associated with readmissions in patients undergoing partial gastrectomy

		No readmission	Index readmission	Non-index readmission	*P value*
	n	3764 (81.9%)	483 (10.5%)	348 (7.6%)	
**Patient Age**	Median Age (IQR)	72 (63-78)	70 (59-77)	72 (63-77)	0.002
	18-56	519 (13.8%)	97 (20.1%)	54 (15.5%)	0.001*
	57-64	522 (13.9%)	71 (14.7%)	45 (12.9%)	
	65-69	501 (13.3%)	62 (12.8%)	31 (8.9%)	
	70-75	810 (21.5%)	95 (19.7%)	95 (27.3%)	
	76+	1413 (37.5%)	158 (32.7%)	123 (35.3%)	
**Sex**	Male	2284 (60.7%)	292 (60.5%)	212 (60.9%)	0.991
	Female	1481 (39.3%)	191 (39.5%)	136 (39.1%)	
**Ethnicity**	White	3249 (86.3%)	414 (85.7%)	300 (86.2%)	0.292
	Asian	138 (3.7%)	27 (5.6%)	15 (4.3%)	
	Non-White Non-Asian	196 (5.2%)	25 (5.2%)	21 (6.0%)	
	Other	182 (4.8%)	17 (3.5%)	12 (3.5%)	
**Deprivation Quintiles**	1	823 (21.9%)	98 (20.3%)	77 (22.1%)	0.961
	2	786 (20.9%)	97 (20.1%)	75 (21.6%	
	3	782 (20.8%)	107 (22.2%)	77 (22.1%)	
	4	689 (18.3%)	86 (17.8%)	61 (17.5%)	
	5	685 (18.2%)	95 (19.7%)	58 (16.7%)	
**Charlson score (without cancer)**	0	2210 (58.7%)	271 (56.1%)	200 (57.5%)	0.257
	1-4	758 (20.1%)	89 (18.4%)	75 (21.6%)	
	5+	797 (21.2%)	123 (25.5%)	73 (21.0%)	
**Volume grouping (use the Average annual operation volume of hospital to split the cohorts equally)**	<=42	664 (17.6%)	95 (19.7%)	41 (11.8%)	<0.001*
	43-64	803 (21.3%)	80 (16.6%)	107 (30.8%)	
	65-74	770 (20.5%)	119 (24.6%)	61 (17.5%)	
	75-97	718 (19.1%)	92 (19.1%)	75 (21.6%)	
	>97	810 (21.5%)	97 (20.1!)	64 (18.4%)	
**Reoperation (within the same admission)**	Yes	89 (2.4%)	*	*	0.449
**Minimal Access Surgery**	Yes	682 (18.1%)	97 (20.1%)	50 (14.4%)	0.103
**Length of stay**	Median LOS(IQR)	10 (8-15)	11 (8-17)	11 (9-16)	0.002*
**90-day mortality**	Yes	89 (2.4%)	24 (5.0%)	17 (4.9%)	<0.001*
**5-year mortality**	Yes	1459 (38.8%)	208 (43.1%)	159 (45.7%)	0.012

Supplementary Table 3- Predictors of readmission post esophagectomy

	Emergency Readmission within 90 days post surgery			
	Odds Ratio	P>z	[95% Conf. Interval]	
**Gender**				
**Male**	1			
**Female**	1.17	<0.001	1.08	1.27
**Age groups (years)**				
**18-56**	1			
**57-64**	0.90	0.031	0.81	0.99
**65-69**	0.86	0.007	0.78	0.96
**70-75**	0.91	0.085	0.82	1.01
**76+**	0.91	0.128	0.80	1.03
**Ethnicity**				
**White**	1			
**Asian**	1.58	0.005	1.15	2.16
**Non-White Non-Asian**	0.92	0.711	0.58	1.46
**Other**	0.74	0.001	0.62	0.89
**Deprivation quintiles**				
**1**	1			
**2**	0.91	0.094	0.81	1.02
**3**	0.89	0.046	0.80	1.00
**4**	0.91	0.087	0.82	1.01
**5**	0.87	0.017	0.78	0.98
**Charlson**				
**0**	1			
**1-4**	1.24	<0.001*	1.14	1.34
**5+**	1.30	<0.001*	1.18	1.43
**Reoperation - No**	1			
**Reoperation - Yes**	1.02	0.780	0.87	1.20
**Minimal Access - No**	1			
**Minimal Access - Yes**	1.07	0.057	1.00	1.15
**Hospital Volume groups**				
**<=42**	1			
**43-64**	1.17	0.011	1.04	1.31
**65-74**	1.06	0.306	0.95	1.20
**75-97**	1.11	0.090	0.98	1.24
**>97**	1.15	0.025	1.02	1.29

Supplementary Table 4- Predictors of readmission post gastrectomy

	Emergency Readmission within 90 days post surgery			
	Odds Ratio	P>z	[95% Conf. Interval]	
**Gender**				
**Male**	1			
**Female**	1.09	0.110	0.98	1.21
**Age groups (Years)**				
**18-56**	1			
**57-64**	0.84	0.054	0.71	1.00
**65-69**	0.78	0.007	0.65	0.93
**70-75**	0.83	0.026	0.71	0.98
**76+**	0.77	0.001	0.66	0.89
**Ethnicity**				
**White**	1			
**Asian**	1.11	0.417	0.86	1.44
**Non-White Non-Asian**	0.94	0.643	0.73	1.22
**Other**	0.72	0.015	0.56	0.94
**Deprivation quintiles**				
**1**	1			
**2**	0.94	0.456	0.81	1.10
**3**	1.06	0.487	0.91	1.23
**4**	1.02	0.792	0.87	1.19
**5**	1.00	0.975	0.85	1.18
**Charlson Comorbidity Index**				
**0**	1			
**1-4**	1.59	0.021	1.02	1.31
**5+**	1.23	0.002	1.08	1.40
**Reoperation - No**	1			
**Reoperation - Yes**	1.07	0.644	0.79	1.45
**Minimal Access - No**	1			
**Minimal Access - Yes**	0.93	0.350	0.80	1.08
**Hospital Volume groups**				
**<=42**	1			
**43-64**	1.08	0.362	0.92	1.27
**65-74**	1.06	0.524	0.89	1.25
**75-97**	1.02	0.781	0.87	1.21
**>97**	1.00	0.958	0.84	1.18
