# Supplementary figures and images for: Predictors and Significance of Readmission after Esophagogastric Surgery: A Nationwide Analysis
Source: Ann Surg Open. 2024 Jan 26;5(1):e363. doi: 10.1097/AS9.0000000000000363 (PMC11175914; doi:10.1097/AS9.0000000000000363)

Supplementary Figure 1- Impact of readmission cause on survival post esophagectomy

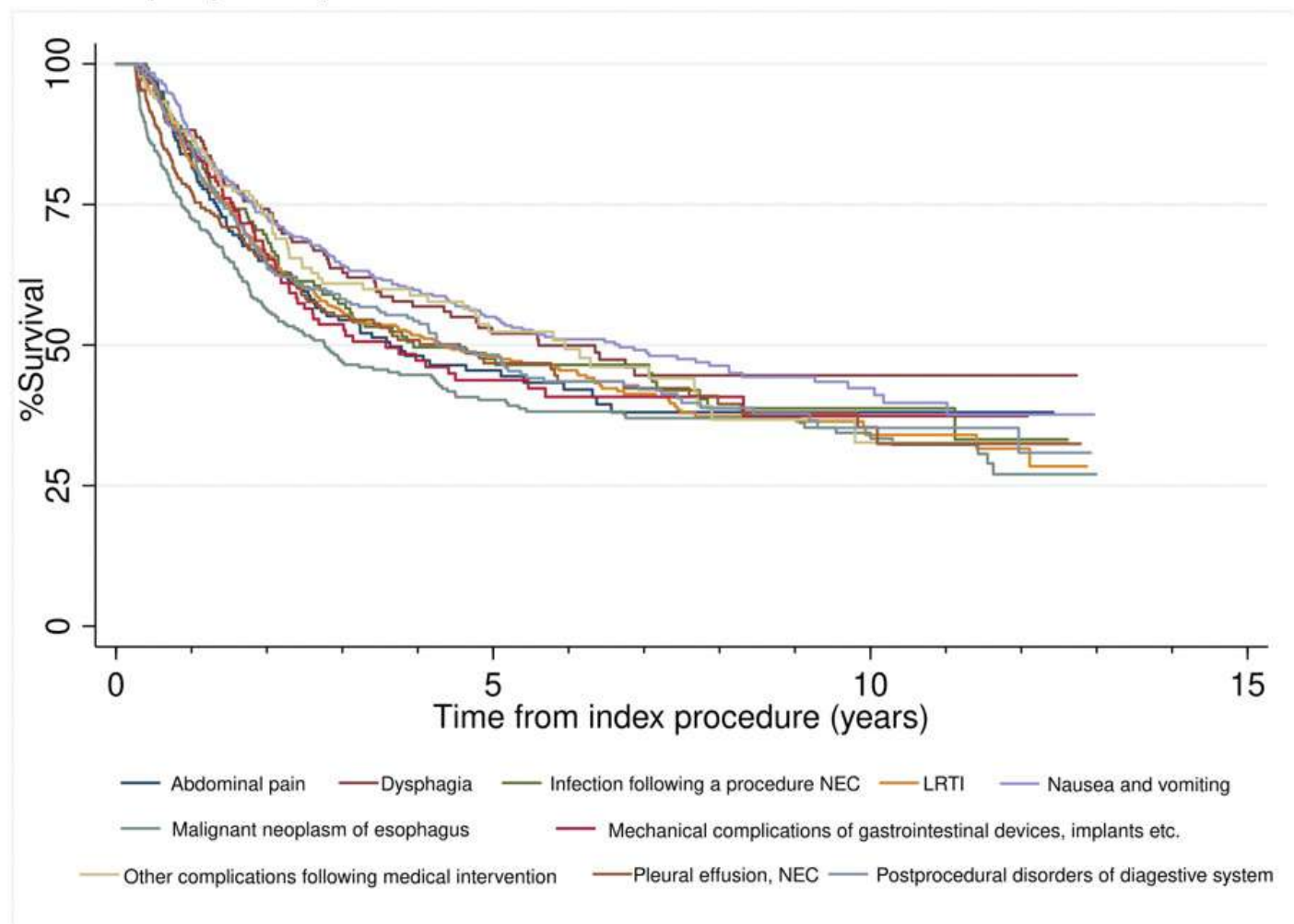

Supplement: Supplementary file 2 [file as9-5-e363-s002.pdf]

Supplementary Figure 2- Impact of readmission cause on survival post gastrectomy

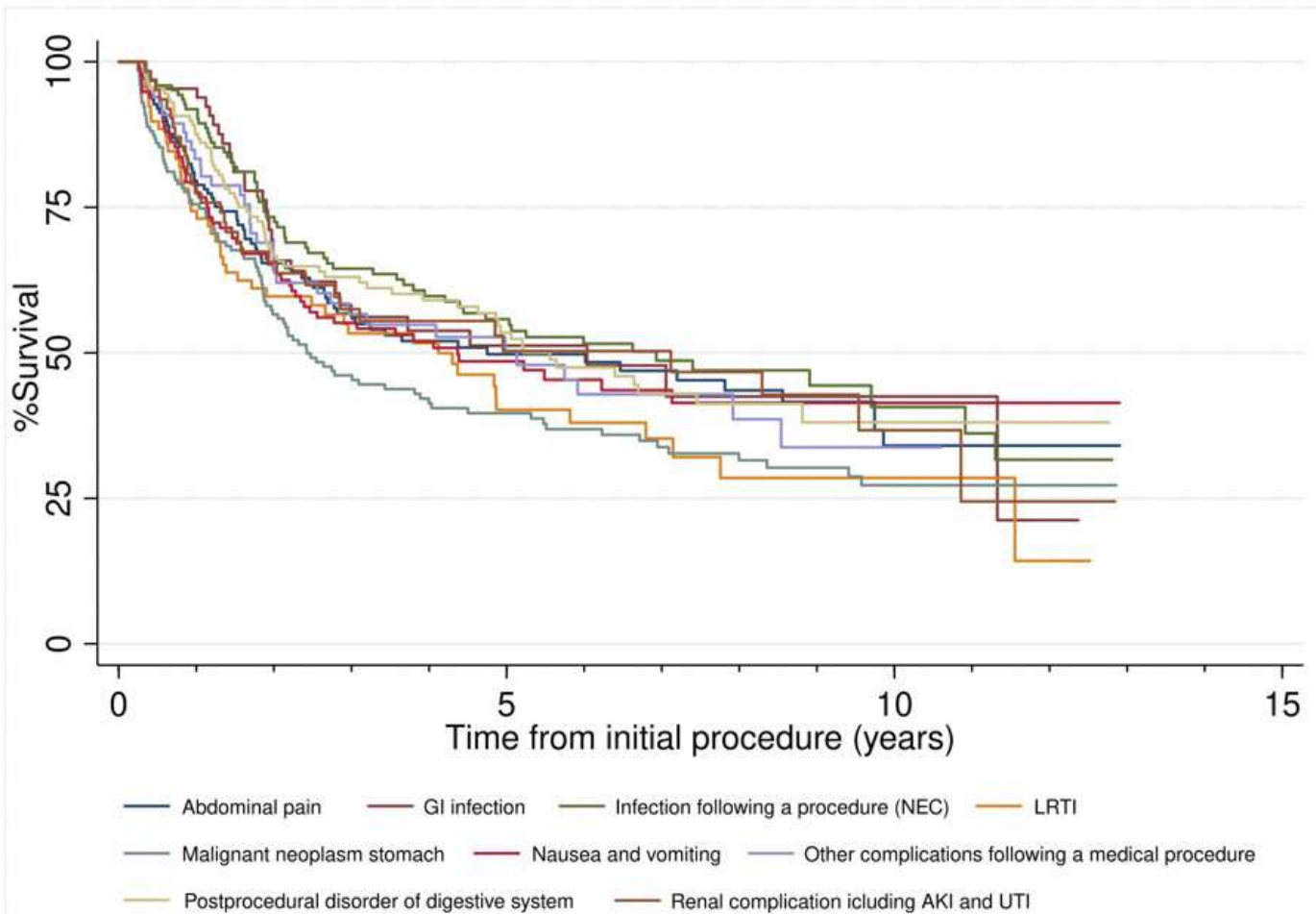

Supplement: Supplementary file 7 [file as9-5-e363-s007.pdf]
