# Supplementary material for: Predictors and Significance of Readmission after Esophagogastric Surgery: A Nationwide Analysis
Source: Ann Surg Open. 2024 Jan 26;5(1):e363. doi: 10.1097/AS9.0000000000000363 (PMC11175914; doi:10.1097/AS9.0000000000000363)

Supplementary Figure 6 – Outcome relationship between hospital volume and 5 year survival post gastrectomy

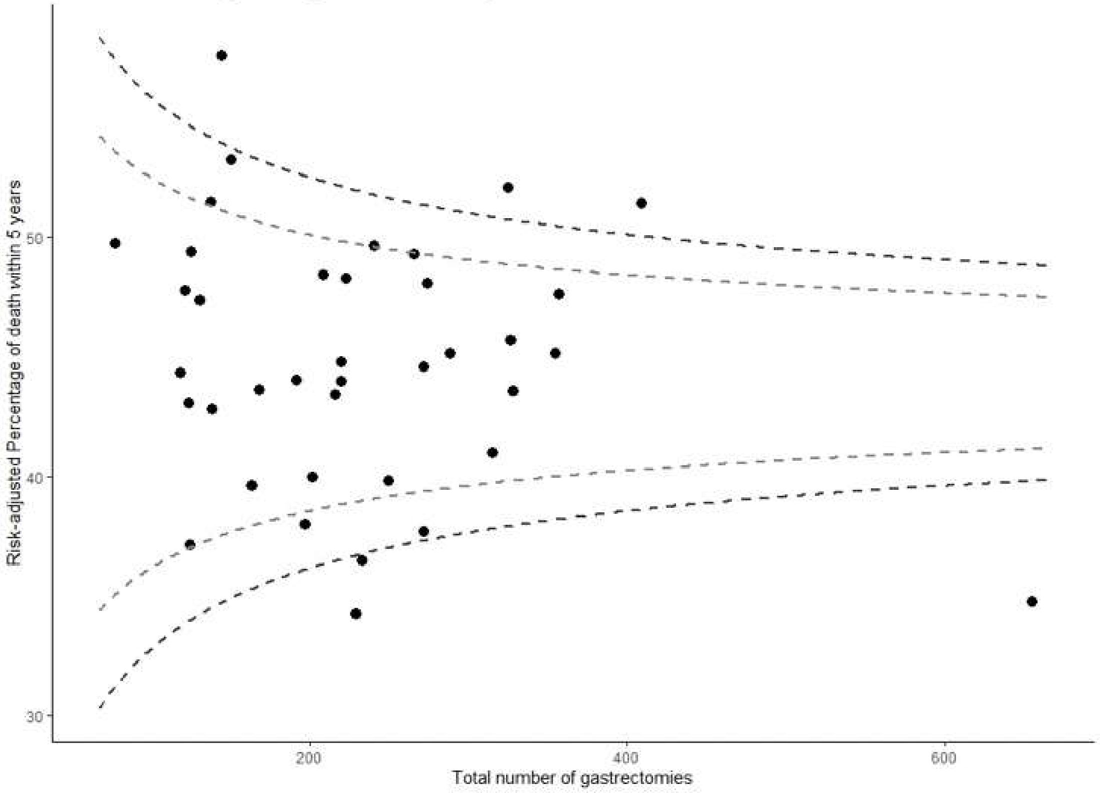

Supplement: Supplementary file 6 [file as9-5-e363-s006.pdf]
